# Allometric scaling of weight to height and resulting body mass index thresholds in two Asian populations

**DOI:** 10.1038/s41387-018-0068-3

**Published:** 2019-01-09

**Authors:** Karoline Hood, Jacob Ashcraft, Krista Watts, Sangmo Hong, Woong Choi, Steven B. Heymsfield, Rajesh K. Gautam, Diana Thomas

**Affiliations:** 1 United States Military Academy, West Point, NY United States; 2 Hanyang University, Seoul, Republic of Korea; 3 Pennington Biomedical Research Center, LSU System, Baton Rouge, LA United States; 4 Center for Quantitative Obesity Research, Montclair State University, Montclair, NJ United States

## Abstract

**Background:**

Body mass index (BMI) represents a normalization of weight to height and is used to classify adiposity. While the capacity of BMI as an adiposity index has been experimentally validated in Caucasians, but there has been little testing Asian populations.

**Methods:**

To determine whether weight scales to height squared in Asian Indians across the general population and in Asian Indian tribes an allometric analysis on the power law model, *W* = *αH*^*β*^, where W is weight (kg) and H is height (m) was performed on cross-sectional weight and height data from India (*N* = 43,880) collected through the Anthropological Survey of India. The database contained males 18–84 years of age spanning 161 districts of 14 states and including 33 different tribes (*N* = 5,549). Models were developed that were unadjusted and adjusted for tribe membership. The Korean National Health and Nutrition Examination Survey (KNHANES) was used to compare to height–weight data from the Anthropological Survey of India and to calculate BMI thresholds for obesity status using a receiver operating characteristic.

**Results:**

The unadjusted power was *β* = 2.08 (*s* = 0.02). The power for the general population (non-tribal) was *β* = 2.11 (*s* = 0.02). Powers when adjusted for tribe ranged from 1.87 to 2.35 with 24 of the 33 tribes resulting in statistically significant (*p* < 0.05) differences in powers from the general population. The coefficients of the adjusted terms ranged from −0.22 to 0.26 and therefore the scaling exponent does not deviate far from 2. Thresholds for BMI classification of overweight in the KNHANES database were BMI = 21 kg/m^2^ (AUC = 0.89) for males 18 kg/m^2^ (AUC = 0.97) for females. Obesity classification was calculated as BMI = 26 kg/m^2^ (AUC = 0.81) and 23 kg/m^2^ (AUC = 0.83) for females.

**Conclusions:**

Our study confirms that weight scales to height squared in Asian Indian males even after adjusting for tribe membership. We also demonstrate that optimal BMI thresholds are lower in a Korean population in comparison to currently used BMI thresholds. These results support the application of BMI in Asian populations with potentially lower thresholds.

## Introduction

Body weight alone cannot characterize human body shape without accounting for stature. As a result, identifying the mathematical formulation that accurately normalizes body weight by height has been of long-standing interest^1^.

Body shape indices in humans were first derived in recognition that weight had to be normalized by some function of height in order to classify adiposity. The Metropolitan Life Insurance Company were the first to classify obesity under the assumption that weight is proportional to height^2^, however, high variance in the classifications were noted. Eventually, by the 1960s, Quetelet’s index, which proposed that weight is proportional to the square of height, was adopted^3^. Eventually, Quetelet’s 1832 conjecture was experimentally verified by Ancel Keys in 1972^1^. Ancel Keys referred to Quetelet’s index as the body mass index (BMI) and his experiment has since then been reproduced in larger sample sizes consisting of predominately Caucasians^4–6^.

BMI relies on the assumption that weight scales to height squared, independent of race and sex. While it is generally accepted that the scaling exponent is 2, the validity of this assumption across races is debated^7^. Moreover, while it is universally applied to classify individuals affected by obesity^6,8–10^, there is much discussion of whether BMI cutoffs to classify excess adiposity are appropriate for use in Asian populations^6,11^. Populations in India exhibit similar characteristics observed in other Asian populations such as higher abdominal adiposity and percent body fat for given BMI in comparison to Caucasian populations^12,13^. This could be due to either (1) BMI being an inappropriate index for Asian populations or (2) that the thresholds classifying excess adiposity from BMI should be lowered^11^. Recently, national survey data have been compiled in some Asian countries permitting more broad analysis of Quetelet’s hypothesis^6^. While previous analysis of this data has been performed using BMI^14^, the analysis has not included testing whether BMI is the correct scaling.

Here, we first analyze the correct normalization of weight and height in the Asian Indian population through an allometric analysis using a large nationally representative Asian Indian database that included measured weights and heights. This database also included tribal populations that are smaller in stature and size compared to the general population. To our knowledge, there has not yet been an allometric analysis between weight and height that extends to tribes. The large Asian Indian database does not include clinical measurements such as cardiometabolic risk factors or body fat. As a result we could not evaluate BMI thresholds in the Asian Indian database. However, we were able to compute BMI thresholds in a Korean database that included measured percent body fat. The validity of BMI as the correct adiposity index along with evidenced based thresholds are important to consider when classifying adiposity and obesity-related co-morbidities in Asian populations.

## Methods

### Study design and rationale

This study was designed to evaluate three questions.

1. Does weight scale to height squared in Asian Indians?
2. Do BMI thresholds to classify obesity differ in Asian populations?
3. Do weight–height relationships differ among Asian populations?

To address Question 1, allometric power law models were developed and the optimal exponent was derived using a large nationally representative database acquired through the Anthropological Survey of India. The second question was examined using a second database, the Korean National Health and Nutrition Examination Survey (KNHANES). Using percent body fat thresholds derived from relationships between percent body fat and cardiometabolic risk in a Korean population^15^, we applied a receiver operating characteristic analysis to determine corresponding optimal BMI thresholds. Finally, we plotted weight to height graphs for general and tribal Asian Indian populations with the Korean data to compare differences in weight to height relationships.

### Participants

#### The Anthropological Survey of India

Data were referenced from two individual national health surveys of India. Our analysis referenced complete data of height and weight measurements of 43,880 adult males age 15–54 years obtained from both surveys.

The Anthropological Survey of India^16^ is a long-standing national effort to study the tribes and other communities that form the population of India both from the biological and cultural point of view. The study sample is based on basic anthropometric data collected on healthy and active adult males between the ages of 18–84 years collected in two surveys; one from 1965 to 1970^17–19^. The survey data have been applied previously to evaluate nutrition and health status differences between tribes, castes, socio-economic status, and geographic region^14,20–22^. All previous analysis assumed that weight scales to height squared. Our application of the survey data tests this assumption. Among various anthropometric variables directly measured and contained in the database, we retained body weight and height for our analysis.

The Anthropological Survey of India included measurements from 34 tribal populations from 14 of the 29 different states in India. The represented states are Maharashtra, Gujarat, Madhya Pradesh, Chhattisgarh, Orissa, Jharkhand, Bihar, Uttar, Pradesh, Haryana, Punjab, Uttaranchal, Jammu & Kashmir, Assam, and Meghalaya. Measurements were additionally collected from non-tribal populations in each state to achieve a representative sample of the population of India. Formal human subject review boards came into existence by the National Research Act of 1974, which post-dates the first survey wave of the Anthropological Survey of India. However, the Anthropological Survey of India housed its own internal review board which considered the protection of human rights. As guided by the internal board, subjects provided verbal informed consent to participate in the survey.

The second survey was conducted by the National Family Health Survey (NFHS) from 2005 to 2006^23^. The NFHS are nationwide surveys performed over a representative sample of households throughout India. The NFHS protocol and consent procedures were approved by the Ethical Committee of International Institute of Population Sciences (IIPS), Mumbai.

#### The Korean National Health and Nutrition Examination Survey (KNHANES)

A nationwide survey that assesses health and nutrition status in Koreans^24^ was administered between 2007–2009 (KNHANES IV) and 2010–2012 (KNHANES V) by the Ministry of Health and Welfare and the Korean Centers for Disease Control and Prevention. KNHANES IV and V contained percent body fat measured by dual energy X-ray absorptiometry (DXA). The KNHANES study protocol was approved by the Korea Centers for Disease Control and Prevention Institutional Review Board, and all subjects provided written informed consent

### Statistical methods

#### Allometric model analysis

All analyses focused on determining the power, *β* in the allometric model *W* = *αH*^*β*^, where *W* represents body weight in kilograms (kg), *H* represents height in meters (m), *β* is referred to as the scaling exponent, and *α* is referred to as the proportionality constant.

The allometric model

$$W = \alpha H^\beta,$$

was log-transformed: ${\mathrm{ln}}(W) = {\mathrm{ln}}\left( {\alpha H^\beta } \right)$. After applying mathematical laws of logarithms the equation transforms to:

1 $$\ln \left( {\mathrm{W}} \right) = \ln \left( {\mathrm{\alpha }} \right) + {\mathrm{\beta }}\ln \left( {\mathrm{H}} \right).$$

Equation (1) represents the functional form where simple linear regression was applied to determine the intercept, $\ln \left( \alpha \right)$, and slope, β. The coefficients were estimated using ordinary least squares (OLS) regression to experimentally yield the value of β that best explains the scaling relationship. The above model was fit to the full dataset of 43,880 participants that had all height and weight measurements and to the dataset of 38,331 participants which excluded tribe members.

Next, a model that adjusts for tribal membership was developed. Specifically, the model was developed to determine whether the scaling exponent was consistent across tribes. The allometric model that accounts for tribe membership is:

$$W = \alpha H^{\beta + {\boldsymbol{\eta T}}},$$

where **T** is a vector of indicator variables representing tribal membership and ${\boldsymbol{\eta }} = \left( {\eta _1,n_2, \ldots ,n_{35}} \right)$ is a vector representing the difference in scaling exponent based on tribal membership. Similar to^1^ we log transform and apply mathematical laws of logarithms to arrive at:

2 $${\mathrm{ln}}(W) = \ln \left( {\mathrm{\alpha }} \right) + {\mathrm{\beta }}\ln \left( {\mathrm{H}} \right) + {\boldsymbol{\upeta}} {\mathbf{T}}\ln \left( {\mathrm{H}} \right).$$

The scaling exponent for an individual from the non-tribal population is β, whereas the scaling coefficient for an individual from tribe *k* is *β* + *η*_*k*_. If **η** is the zero vector, the scaling coefficient is constant across all populations (both all tribes and for those with no tribal heritage).

#### Receiver operating characteristic curve analysis

A ROC analysis was performed on the KNHANES dataset using the statistical program R (**R** Core Team (2013)). The R package dplyr was used to group and filter the KNHANES data by gender. Percent body fat cutoffs were set to 17% to classify overweight and 32% to classify overweight for males and females, respectively^15^. For obesity classifications, body fat cutoffs were set to 21% for overweight and 37% for males and females, respectively^15^. The cutoff values were determined from relationship between percent body fat and cardiometabolic risk factors in an epidemiological study conducted in Korea^15^. Binary outputs were assigned as 0 if percent body fat was below the cutoff value and 1 if the percent body fat cutoff was above the cutoff value. The R package, pROC was then used to classify true positives, false positives, true negatives, and false negatives where BMI is used to classify percent body fat. The pROC package outputs the optimal threshold, which simultaneously maximizes true positives and minimizes false negatives and the resulting area under the curve (AUC) of the ROC curve. Ninety-five percent confidence intervals for the AUC and thresholds were also provided by the pROC package.

### Code availability

R script code used for statistical analysis will be provided upon request by the contributing author.

## Results

### Participants

From the entire database, a total of *N* = 43,880 participants had all necessary variables for model development. Of these, *N* = 5549 were members of various tribes and 38,331 were from different castes and religious groups. Subject characteristics for the each tribe, the non-tribe population and total population appear in Table 1. The KNHANES dataset is also described in Table 1.

**Table 1:** Means ± SD of weight (kg), height (m), and age (years) in participants used for allometric model analysis

Name (*N*)	Weight (kg)	Height (m)	Age (years)
Agaria (150)	53.57 ± 6.05	1.66 ± 0.06	36.47 ± 9.66
Andh (49)	45.49 ± 4.08	1.61 ± 0.05	34.47 ± 10.88
Banhara (99)	45.99 ± 5.18	1.63 ± 0.05	35.21 ± 10.33
Bhil (846)	47.48 ± 5.92	1.62 ± 0.06	32.78 ± 10.45
Bhuiya (246)	46.68 ± 5.19	1.58 ± 0.06	34.69 ± 9.41
Dubla (50)	45.40 ± 5.32	1.61 ± 0.06	29.02 ± 8.49
Gond (1103)	48.72 ± 5.46	1.62 ± 0.06	35.62 ± 10.32
Ho (50)	45.64 ± 5.39	1.60 ± 0.04	32.14 ± 8.41
Kachari (148)	53.22 ± 5.78	1.62 ± 0.05	35.60 ± 11.26
Karan (200)	51.11 ± 7.80	1.63 ± 0.06	34.63 ± 9.20
Kathodi (50)	44.40 ± 3.31	1.62 ± 0.05	30.88 ± 8.53
Khond (100)	46.77 ± 4.59	1.56 ± 0.05	35.81 ± 9.59
Koch (150)	50.59 ± 6.97	1.63 ± 0.06	35.37 ± 12.59
Kol (200)	48.76 ± 5.01	1.61 ± 0.06	36.60 ± 10.26
Korku (150)	47.95 ± 5.00	1.62 ± 0.05	37.24 ± 10.89
Korwa (51)	50.24 ± 6.17	1.55 ± 0.07	33.61 ± 9.36
Lalung (49)	49.29 ± 6.22	1.60 ± 0.07	34.16 ± 9.23
Mahadeokoli (100)	48.37 ± 5.74	1.63 ± 0.06	37.35 ± 10.34
Majhi (50)	48.76 ± 5.05	1.58 ± 0.06	33.52 ± 11.14
Mech (50)	52.72 ± 4.30	1.60 ± 0.04	37.90 ± 11.08
Miri (50)	49.64 ± 5.11	1.59 ± 0.06	36.94 ± 12.16
Munda (148)	46.97 ± 4.55	1.58 ± 0.06	33.97 ± 10.32
Oraon (298)	48.22 ± 6.07	1.61 ± 0.06	31.55 ± 10.28
Paroja (50)	44.28 ± 4.64	1.60 ± 0.06	33.56 ± 6.82
Pnarkhaski (49)	49.33 ± 4.60	1.58 ± 0.06	31.45 ± 11.35
Rabari (50)	55.66 ± 10.68	1.66 ± 0.07	31.18 ± 7.18
Sahariva (204)	48.12 ± 5.46	1.63 ± 0.06	32.26 ± 9.73
Santal (347)	47.04 ± 5.32	1.61 ± 0.05	34.13 ± 10.73
Santhal (106)	47.00 ± 4.71	1.60 ± 0.05	32.47 ± 9.68
Savara (200)	46.50 ± 4.66	1.59 ± 0.06	33.84 ± 8.81
Sonr (56)	46.43 ± 5.67	1.62 ± 0.07	32.11 ± 9.44
Tharu (50)	53.36 ± 6.92	1.65 ± 0.05	28.84 ± 10.39
Warli (50)	43.32 ± 4.41	1.60 ± 0.06	32.50 ± 10.38
Non-tribal (38,331)	50.61 ± 7.89	1.64 ± 0.06	34.24 ± 11.04
Total Anthropological Survey of India (43,880)	50.30 ± 7.71	1.64 ± 0.06	34.24 ± 10.96
KNHANES males (3849)	69.34 ± 10.62	1.70 ± 6.58	47.24 ± 16.30
KNHANES females (5089)	57.20 ± 8.82	156.99 ± 6.44	47.50 ± 16.19

### Scaling of body mass to height

The unadjusted power, *β* from model (1) was 2.08 (*s* = 0.02, *R*^2^ = 0.30). After adjusting for tribe (Table 2), *β* = 2.09 (*s* = 0.02, *R*^2^ = 0.31)). When tribal-based adjustments to the scaling coefficient are considered in the second model, we find that most of the tribes have a statistically different power compared to the general, non-tribal, population (24 of 33 with *p* < 0.05). The range of the coefficient of the tribal interaction term was −0.22 to 0.26. The estimated scaling exponents ranged from 1.86 (for the Warli tribe) to 2.35 (for the Korwa tribe). Consideration of the general non-tribal population separately resulted in an exponent of 2.11 (*s* = 0.02, *R*^2^ = 0.30).

**Table 2:** Model coefficients for tribe inclusion term, ${\mathrm{ln}}(W) = \ln \left( {\mathrm{\alpha }} \right) + {\mathrm{\beta }}\ln \left( {\mathrm{H}} \right) + {\boldsymbol{\eta }}{\mathbf{T}}{\mathrm{ln}}({\mathrm{H}})$. The scaling exponent was *β* = 2.09 (*s* = 0.02)

Tribe	*η*
Agaria	0.071 (*s* = 0.020)
Andh	−0.138 (*s* = 0.037)
Banjara	−0.153 (*s* = 0.025)
Bhil	−0.081 (*s* = 0.009)
Bhuiya	0.018 (*s* = 0.017)
Dubla	−0.139 (*s* = 0.008)
Gond	−0.021 (*s* = 0.008)
Ho	−0.106 (*s* = 0.037)
Kachari	0.159 (*s* = 0.021)
Karan	0.032 (*s* = 0.018)
Kathodi	−0.195 (*s* = 0.036)
Khond	0.057 (*s* = 0.027)
Koch	0.041 (*s* = 0.021)
Kol	0.009 (*s* = 0.018)
Korku	−0.057 (*s* = 0.021)
Korwa	0.256 (*s* = 0.039)
Lalung	0.061 (*s* = 0.037)
Mahadeokoli	−0.060 (*s* = 0.025)
Majhi	0.087 (*s* = 0.38)
Mech	0.201 (*s* = 0.037)
Miri	0.095 (*s* = 0.037)
Munda	0.007 (*s* = 0.022)
Oraon	−0.024 (*s* = 0.015)
Paroja	−0.171 (*s* = 0.037)
Pnarkhasi	0.137 (*s* = 0.038)
Rabari	0.120 (*s* = 0.034)
Sahariya	−0.061 (*s* = 0.018)
Santal	−0.059 (*s* = 0.014)
Santhal	−0.044 (*s* = 0.025)
Savara	−0.025 (*s* = 0.019)
Sonr	−0.118 (*s* = 0.034)
Tharu	0.093 (*s* = 0.035)
Warli	−0.220 (*s* = 0.037)

### Body mass index thresholds for KNHANES

Obesity and overweight classification thresholds for BMI classification, corresponding AUC and 95% confidence intervals derived from the KNHANES database appear in Table 3. The thresholds for overweight were 22 and 18 kg/m^2^ for males and females, respectively. For obesity, the thresholds were 26 and 23 kg/m^2^ for males and females.

**Table 3:** The optimal BMI thresholds that meets overweight and obesity percent body fat cutoffs related to cardiometabolic risk (15) in Koreans. The AUC and 95% confidence intervals are provided

Classification	BMI threshold	AUC
Overweight (males)	21.65 [21.06, 22.32]	0.89 [0.87, 0.91]
Obesity (males)	25.93 [24.87, 26.08]	0.81 [0.79–0.83]
Overweight (females)	18.22 [16.55, 19.86]	0.97 [0.94–1.00]
Obesity (females)	23.20 [23.09, 23.75]	0.83 [0.82, 0.84]

### Comparison of weight to height in general Asian Indian, tribal Asian Indian, and Korean populations

**Fig. 1:**
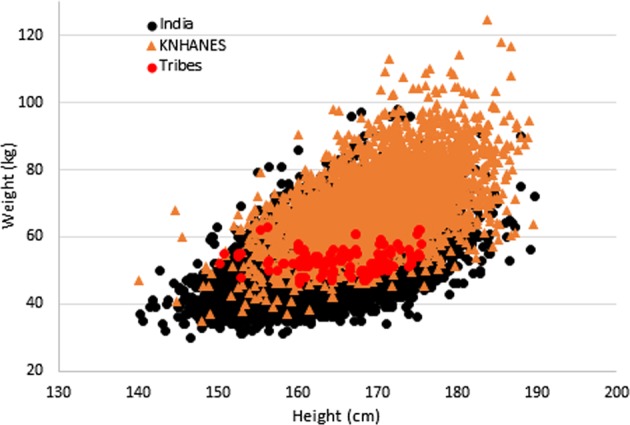
Overlay of weight (kg) versus height (cm) for the general Asian Indian population (black circles), the Korean population (tan triangles), and the tribal Asian Indian populations (red circles). The Asian Indian population contains data of individuals smaller in stature and size than the Korean population. The tribal populations appear to range in height yet be consistently smaller in weight in comparison to the general Asian Indian and Korean populations

Figure 1 provides an overlay of weight versus height in the general population Asian Indian (solid black circles), tribal Asian Indian (solid red circles), and male Korean populations (solid tan triangles). While there was overlap in the data, the general Asian Indian population also consisted of smaller size and stature individuals in comparison to KNHANES. The tribal population did not appear to differ in height, but had consistently had smaller body weight.

## Discussion

Here we experimentally confirmed that weight scales to height squared in males from India. We also, for the first time, confirmed that populations, while smaller in stature and size, essentially also scale to height squared. This analysis was made possible by utilizing a unique dataset collected over a span of several decades through the Anthropological Survey of India. Our results justify the application of BMI as the appropriate index that normalizes weight by height in Asian Indians. A second national dataset in Koreans was used to derive BMI thresholds linked to percent body fat cutoffs in a Korean population that has been related to cardiometabolic risk factors^15^. The derived BMI thresholds to classify obesity status were less than those currently employed by the World Health Association^25^.

A major strength of our study was the application of the national survey data in Asian Indians. Determining powers for mass-height scaling laws depends on the availability of a large and diverse database that includes measured height and body mass. The pooled database of body mass and height in Asian Indians used in our analysis spanned geographic regions across India, included various socio-economic strata, consisted of different castes and religions, and included tribal populations.

Our BMI power-scaling results are consistent with the existing literature^4–6^. A recent study found that weight scales to height squared in a Korean population using the Korean National Health and Nutrition Examination Survey (KNHANES)^6^. The same study also found that after adjusting for race in the United States NHANES, the optimal scaling exponent was also 2.

The derived BMI thresholds in Koreans are also consistent with the literature^26,27^. A threshold of BMI of 22 kg/m^2^ and 26 kg/m^2^ classified overweight and obesity, respectively. A recent joint World Health Organization group recommended that 23 kg/m^2^ (overweight) and 25 kg/m^2^ (obesity) be applied as BMI thresholds in Asians^28^. However, the thresholds we calculated for females were lower; 18 kg/m^2^ and 23 kg/m^2^, respectively. Similar gender differences in BMI thresholds in Asian populations were reported in the World Health Organization report^11^.

The same World Health Organization committee also reported high variance in BMI thresholds between Asian populations^11^. Although our Asian Indian database did not include clinical body fat measurements or cardiometabolic risk factors measurements, and therefore we could not directly compare BMI thresholds between Asian Indian and Korean populations, we could compare weight for height plots between both populations. The overlay of weight to height demonstrates differences in body weight relative to height. There were overlapping regions between the two populations, however, the Asian Indian population had low weight for height regions that had no overlap with the Korean population. Likewise, the Korean population had high weight for height regions that had no overlap with the Asian Indian population. These differences can be explained by body weight, namely that a subgroup of the Asian Indian population has smaller body weight than the majority of the Korean population and a subgroup of the Korean population has higher body weight than the majority of the Asian Indian population. Additional body shape measures that assess regional adiposity, like waist and hip circumference could be included in national health surveys to understand why BMI thresholds related to risk factors may differ between populations.

### Study limitations

While our study has a number of strengths, our analyses also has several key limitations. First, the Anthropological Survey of India did not include women in their measurements. Collecting data from women in India remains challenging due to long-standing cultural norms. As found in the other studies^6^, we anticipate gender contributing to differences in unadjusted exponent values and this needs to be thoroughly examined with analysis from modern data samples. Our study was also limited due to lack of clinically rigorous body composition measurements such as those included in the US and Korean NHANES protocol prohibiting the evaluating the role of adiposity in scaling exponent. Unfortunately, without measured percent body fat or cardiometabolic risk factors or mortality data^29^, we are unable to evaluate appropriate BMI thresholds in the Asian Indian population and compare these thresholds to those adopted by the US Centers for Disease Control and the World Health Organization^11^. Finally, the US and Korean NHANES protocols follow strict clinical protocols and conduct their evaluations using mobile exam centers. Participants in the Anthropological Survey of India were not weighed in a clinic or in standardized clothing. Unfortunately, there is not currently a practice in India for collecting national health data using mobile clinics like those used by the United States national data collection efforts. Despite these limitations, the Asian Indian dataset still represents the only large and comprehensive database from India which includes tribes.

## Conclusions

This study provides evidence that the appropriate scaling exponent for Asian Indians is 2, supporting the use of BMI as the appropriate normalization of weight by height in Asian Indians. BMI thresholds in a Korean population were determined lower than currently applied BMI thresholds for obesity classification. This supports the use of country and race-specific BMI thresholds for classifying obesity in Asian populations.
